# Partial Protection From Lupus-Like Disease by B-Cell Specific Type I Interferon Receptor Deficiency

**DOI:** 10.3389/fimmu.2020.616064

**Published:** 2021-01-08

**Authors:** Emma J. Keller, Neeva B. Patel, Madeline Patt, Jane K. Nguyen, Trine N. Jørgensen

**Affiliations:** ^1^ Department of Inflammation and Immunity, Lerner Research Institute, Cleveland Clinic Foundation, Cleveland, OH, United States; ^2^ Cleveland Clinic Lerner College of Medicine, Dept. of Molecular Medicine, Case Western Reserve University, Cleveland, OH, United States; ^3^ Robert J. Tomsich Pathology and Laboratory Medicine Institute, Cleveland Clinic, Cleveland, OH, United States

**Keywords:** lupus, type I interferon, germinal center B cell, Bcl2, autoantibody, B cell, Type I interferon receptor

## Abstract

Systemic lupus erythematosus (SLE) is an autoimmune disease that can present with many different permutations of symptom presentation. A large subset of SLE patients have been shown to present with elevated interferon stimulated gene (ISG) expression, and Type I IFNs (IFNαβ) have been shown to drive disease in murine models through global IFNα Receptor (IFNAR) knockouts. However, the disease contribution of distinct immune cell subsets in response to constitutively increased levels of IFNαβ is not fully understood. We utilized a B-cell specific IFNAR knockout (BΔIFNAR) on the B6.Nba2 spontaneous-lupus background to determine the contribution of IFNαβ stimulated B cells in disease. We found that IFNαβ signaling in B cells is driving increased splenomegaly, increased populations of activated B cells, and increased populations of germinal center (GC) B cells, memory B cells, and plasma blasts/cells, but did not affect the development of glomerulonephritis and immune-complex deposition. IFNAR expression by B cells also drove production of anti-chromatin IgG, and anti-dsDNA and -nRNP IgG and IgG_2C_ auto-antibody levels, as well as increased *Bcl2* expression, affecting GC B cell survival in B6.Nba2 mice.

## Introduction

Systemic Lupus Erythematosus (SLE) is a chronic autoimmune disease. SLE patients have a lower quality of life when compared to that of patients with other chronic illnesses (1). Treatment of SLE has advanced in the recent past with the FDA approval of Belimumab in 2011, the first SLE drug approved in 50 years (2). However, one remaining critical obstacle in the field is the heterogeneity of symptom presentation between SLE patients. For physicians using the American College of Rheumatology SLE diagnosis criteria, there are 330 different permutations of symptom presentations that can be diagnosed as SLE (3). As the field moves toward more targeted treatment strategies, it is imperative to understand which presentations of SLE could be treated with a particular therapy, and which presentations are not likely to respond to the therapy.

It has been known for decades that many SLE patients present with elevated levels of type I interferons (IFNαβ) (4–6). In 2003, multiple publications showed that SLE patient PBMCs had a significant increase in IFNαβ-stimulated gene expression (7–10). Combined with the knowledge that interferon treatment for non-SLE conditions may lead to the development of auto-immune responses and SLE symptoms (11, 12), interferons quickly became an area of study in SLE. As such, blocking the type I IFN receptor (IFNAR) was shown to inhibit IFNαβ production and decrease the development of plasma cells in patients (13, 14). Furthermore, according to data presented at the 2020 EULAR E-Congress of Rheumatology, phase III trials of Anifrolumab, a monoclonal antibody that blocks type IFNAR, have been successful in reducing flares and BICLA (BILAG-based Combined Lupus Assessment) scores in patients (14–16).

The role of IFNαβ in SLE has been confirmed by lupus-like disease amelioration in global IFNAR knockouts on a multitude of murine lupus backgrounds (17–21). In the B6.Nba2 spontaneous lupus mouse model, global IFNAR deficiency leads to decreased autoantibody production, a reduction in serum IgG, reduced splenic *Ifi202* expression and decreased splenomegaly (17). Similarly, IFNAR-deficient New Zealand Black (NZB) mice displayed significantly reduced auto-antibodies and a significant reduction in splenomegaly (19, 22). However, despite the overwhelming evidence supporting blocking IFNAR in SLE, the fact that IFNAR is expressed by most cells of the body and stimulation contributes a wide array of anti-viral responses, suggests that IFNAR blockade could have unwanted side effects. Moreover, as not all patients present with elevated IFNαβ levels, it is unlikely that such therapy will benefit all patients. To determine which aspects of disease and symptom presentation are IFNαβ-driven through B cells, we generated B cell-specific IFNAR-/- lupus-prone B6.Nba2 mice (BΔIFNAR). Mice were followed for four months to determine the primary effects of B cell-specific IFNAR-deficiency on cellular and serological measures of lupus-like disease observed in B6.Nba2 mice.

## Materials and Methods

### Mice

B6(Cg)-*Ifnar1^tm1.1Ees^*/J (The Jackson Laboratories, strain #028256) and B6.C(Cg)-*Cd79a^tm1(cre)Reth^*/EhobJ (C57BL6.Mb1-cre) (The Jackson Laboratories, #020505) were crossed for generation of B6.BΔIFNAR mice or each backcrossed onto the B6.Nba2.ABC background (B6.Nba2) for subsequent generation of B6.Nba2.IFNAR^flx/flx^MB1^cre/+^ (B6.Nba2.BΔIFNAR) mice. The presence of each transgene was determined by PCR as previously described (23, 24). Primers used were: *IFNAR5′*: 5*′* TGC TTT GAG GAG CGT CTG GA 3*′ IFNAR3′*: 5*′* CAT GCA CTA CCA CAC CAG GCT TC 3*′ IFNARΔ5′*: 5*′* TAG CCC CAG GGT AGT TAA CTC TTG A 3*′ MB1cre F*: 5*′* CCC TGT GGA TGC CAC CTC 3*′*, *MB1cre R*: 5*′* GTC CTG GCA TCT GTC AGA G 3*′*. In order to determine the presence of the NZB-derived *Nba2* locus PCR analysis was done for the presence of NZB-derived D1mit36, D1mit47, D1mit113, and D1mit209 markers as previously described (25). All mice were born and housed in specific pathogen-free housing at Lerner Research Institute. Only female mice were used for this study. All animal research were approved and performed in accordance with the institutional guidelines.

### Organ Harvest and Processing

B6, B6.BΔIFNAR, B6.Nba2.BΔIFNAR and B6.Nba2 female mice were harvested at 4 months of age in several separate cohorts. Spleens were harvested and weighed. A middle portion of the spleen was frozen in OCT for immunohistochemistry. A small portion of the spleen was made into a single cell suspension in 1x phosphate buffered saline (PBS) pH7.4 and incubated at 37°C for 10 mins for isolation of spleen supernatant and single cells for RNA extraction. The remaining spleen was similarly processed into single cells after which red blood cells (RBCs) were lysed using ACK (Ammonium-Chloride-Potassium) lysis buffer (0.15 M NH4Cl, 0.01 M KHCO3, 0.2 mM EDTA, pH 7.3) and the cells were used for flow cytometry. Bone marrow from the left femur and left tibia was flushed out with 1x PBS and used for flow cytometry after RBC lysis as described above. One kidney was removed and dissected into two coronal sections. One half was preserved in OCT, the other half was fixed in 10% formalin.

### IFNαA Stimulation of Splenocytes for Detection of Ifit2

Splenocytes were isolated from B6.Nba2.BΔIFNAR and B6.Nba2 mice and stimulated with 1000 u/mL of recombinant mouse IFN-αA (Fisher Scientific, Hampton, NH, USA) in primary cell media (RPMI w/L-glutamine, 10% fetal bovine serum, 1% Hepes, 1% non-essential amino acids, and 1% streptomyocin) and incubated overnight. Upon harvest, cells were stained for surface markers of B cells, T cells, pDCs and cDCs, fixed and permeabilized using the Foxp3 transcription factor staining buffer set (Invitrogen, Carlsbad, CA, USA), and stained intracellularly with a rabbit anti-mouse Ifit2 antibody (26, 27) (kind gift by Dr. Sen) and a donkey anti-rabbit IgG Alexa Fluor 647-conjugated secondary antibody (eBiolegend Clone Poly4064). Levels of Ifit2 expression was determined by flow cytometry.

### Flow Cytometry

Flow cytometry for splenocytes and bone marrow was run on a BD LSR Fortessa™ flow cytometer (BD Biosciences, San Jose California, USA) and data was collected using BD FACSDiva™ software (BD Biosciences, San Jose California, USA). Data were analyzed using FlowJo Version 10 Software (FlowJo, Ashland, Oregon, USA). The following antibodies were used for staining and sourced from eBioscience (San Diego, CA) unless otherwise noted. Antibodies with the following specificities were used for staining: B220 Clone RA3-6B2, CD3 Clone 145-2C11, CD4 Clone GK1.5, CD11b Clone M1/70, CD11c Clone N418, CD16/32 Clone 93, CD19 Clone 1D3, CD21/35 Clone 4E3, CD23 Clone B3B4, CD38 Clone 90, CD40 Clone HM40-3, CD43 Clone R2/60, CD69 Clone H1.2F3, CD93 Clone AA4.1, CD138 Clone 281-2, CXCR5 Cat:551960 (BD Pharmigen), F4/80 Clone BM8, GL7 Clone GL-7, IgD Clone 11-26c, IgM Clone 11/41, PCDA Clone 927, PD-1 Clone J43, Siglec H Clone 440c, SignR1 Clone 22D1. Streptavidin Conjugated Antibodies were used with catalog numbers: 45-4317-82, 12-4317-87 (eBiosciences), and 405208 (Biolegend). Where indicated, specific cell subsets were isolated by cell sorting on a BD FACSAriaII. All cell subsets were gated as shown in **Supplemental Figure 1**.

### Kidney Immunohistochemistry

Formalin-fixed kidneys were transferred to 80% ethanol and embedded in paraffin. Three µm sections were cut and stained with hematoxylin/eosin (Histology Core, Lerner Research Institute) or Periodic acid shiff (PAS) (Thermo Scientific, Waltham, MA, USA) for detection of kidney morphology, damage and cellular infiltrates. Sections were scored in a blinded fashion by a renal pathologist (JN) at the Cleveland Clinic. Kidneys were evaluated on a scale of 0–5 for mesangial and endothelium hypercellularity. Glomerular area was calculated by measuring the area of 5–15 individual glomeruli per section for each mouse. Immunofluorescence was performed on 5µm sections of kidneys preserved in OCT™ (Scigen, Paramount, CA, USA). Sections were thawed, fixed in acetone, blocked with 10% normal goat serum, and stained using Texas-Red conjugated anti-IgG or anti-IgM antibodies (SouthernBiotech, Birmingham, AL, USA) and FITC-conjugated anti- Complement factor C*′*3 antibodies (Immunology Consultants Laboratory Inc., Portland, OR, USA). Imaging was done on a Keyence BZ-X700 All-in-one microscope (Keyence, Osaka, Osaka, Japan) and images were quantified using the Keyence BZ-X analysis software (Keyence, Osaka, Osaka, Japan). A total of 5–15 glomeruli were quantified for each section and the average calculated per mouse.

### Spleen Immunohistochemistry

For immunofluorescent staining of spleens, 5µm sections were cut of spleens preserved in OCT™ compound. On day one of staining, sections were thawed, fixed in acetone, blocked with 10% normal goat serum, and stained using FITC conjugated anti-B220 IgG2a (eBioscience, San Diego, CA, USA) and biotin conjugated anti-GL-7 IgM antibodies (eBioscience, San Diego, CA, USA) followed by Texas-Red conjugated streptavidin (Southern Biotech, Birmingham, AL, USA). Imaging and quantification was done using the same process as for kidney immunofluorescence.

### Quantitative Reverse-Transcriptase-PCR (qRT-PCR)

RNA was isolated from frozen splenocytes using the RNeasy mini kit (Qiagen, Valencia, CA, USA) and quantified using nanodrop technology (Nanodrop ND-1000 Spectrophotometer, Thermo Fisher). Complementary DNA (cDNA) was made using qScript™ DNA supermix (Quanta BioSciences, Gaithersburg, MD) and quantified using nanodrop technology and diluted for qPCR. qPCR was performed with 100ng cDNA using SYBR™ green PerfeCTa^®^ SYBR^®^ Green FastMix^®^ ROX (Quanta BioSciences) and run on a Step One Plus real time PCR system (Applied Biosciences, Foster City, CA, USA). All transcripts were analyzed using β-actin expression as a control. Primers (Integrated DNA Technologies, Skokie, IL, USA) used are listed below:

qPCR primers; *β-actin F*: 5*′* TGG GAA TGG GTC AGA AGG AC 3*′β-actin R*: 5*′* GGT CTC AAA CAT GAT CTG GG 3*′*, *Mx1 F*: 5*′* TTC CTG AAG AGG CGG CTT T 3*′ Mx1 R*: 5*′* GGT TAA TCG GAG AAT TTG CCA A 3*′*, *IL-1β F*: 5*′* CCC TGC AGC TGG AGA GTG TGG A 3*′ IL-1β R*: 5*′* CTG AGC GAC CTG TCT TGG CCG 3*′*, *Bcl6 F:* 5*′* CCT GAG GGA AGG CAA TAT CA 3*′ Bcl6 R:* 5*′* CGG CTG TTC AGG AAC TCT TC 3*′*, *Il6 F:* 5*′* ACA CAT GTT CTC TGG GAA ATC GT 3*′ Il6 R:* 5*′* AAG TGC ATC ATC GTT GTT CAT ACA 3*′*, *Il21 F:* 5*′* CGC CTC CTG ATT AGA CTT CG 3*′ Il21 R:* 5*′* TGG GTC TCC TTT TCT CAT ACG 3*′*, *Blimp F:* 5*′* TAG ACT TCA CCG ATG AGG GG 3*′ Blimp R:* 5*′* GTA TGC TGC CAA CAA CAG CA 3*′*, *Baff F:* 5*′* CAG CGA CAC GCC GAC TAT AC 3*′ Baff R:* 5*′* CCT CCA AGG CAT TTC CTC TTT T 3*′*, *Irf4 F:* 5*′* GCC CAA CAA GCT AGA AAG 3*′ Irf4 R:* 5*′* TCT CTG AGG GTC TGG AAA CT 3*′*, *Ifi202* F: 5*′* GGT CAT CTA CCA ACT CAG AAT 3*′ Ifi202* R: 5*′* CTC TAG GAT GCC ACT GCT GTT G 3*′*, *Bcl2 F 5′:* TGA GTA CCT GAA CCG GCA TCT *Bcl2 R 5′:* GCA TCC CAG CCT CCG TTA T, *Bim F 5′* CGG ATC GGA GAC GAG TTC A *3′ Bim R 5′* TTC CAG CCT CGC GGT AAT CA, *Bclxl F 5′:* TGG AGT AAA CTG GGG GTC GCA TCG *Bclxl R 5′:* AGC CAC CGT CAT GCC CGT CAG G.

### Enzyme-Linked Immunosorbent Assay

#### IgG/IgM/IgA/IgG Subsets

Immunolon 2HB 96 well flat bottom microtiter plates (Thermo Scientific, Waltham, MA, USA) were coated with unlabeled Ig (1010-01) (Southern Biotech, Birmingham, AL, USA) and blocked the following day with 5% gelatin in 1x PBS. Serum samples were diluted to 1:100,000 for IgM and IgG, 1:50,000 for IgA, IgG1, and IgG3, and 1:100,000 for IgG2b and IgG2c and plated in duplicate. HRP-conjugated secondary antibodies specific to IgG (cat#: 1032-05), IgM (cat#: 1020-05), IgA (cat#: 1040-05) IgG_1_ (cat#: 1070-05), IgG_2b_ (cat#: 1090-05), IgG_2c_ (cat#: 1079-05), and IgG_3_ (cat#: 1100-05) (Southern Biotech, Birmingham, AL, USA) were added. Plates were developed with 3,3′,5,5′-Tetramethylbenzidine (TMB) substrate kit (Thermo Scientific, Waltham, MA, USA) and absorbance OD_450nm_ was read using a Victor 3 plate reader (Perkin Elmer, Waltham, MA, USA).

#### Anti-dsDNA and Anti-nRNP IgG Enzyme-Linked Immunosorbent Assay

ELISA was performed using the mouse anti-dsDNA IgG and anti-nRNP IgG ELISA kits (Alpha Diagnostics International, San Antonio, TX, USA) according to the manufacturer’s instructions. Samples were diluted 1:100. IgG subtype ELISAs were done similarly, but developed with anti-IgG subtype specific-HRP antibodies (IgG_1_ (anti-dsDNA only), IgG_2b_, IgG_2c_, IgG_3_)(Southern Biotech).

#### Anti-Chromatin IgG Enzyme-Linked Immunosorbent Assay

2HB plates (Immunlon, Thermo Fisher Scientific, Waltham, MA, USA) were coated with purified chromatin overnight as previously described (17). The following day plates were blocked with 5% gelatin in PBS for ≥2 h. Serum samples were diluted 1:300 in serum diluent (0.5% γ-globulins and 5% gelatin in 1x PBS tween). HRP-conjugated anti-IgG antibody (Southern Biotech, Birmingham, AL, USA) was added and plates were developed with TMB substrate kit (Thermo Scientific, Waltham, MA, USA) and OD_450nm_ was read using a Victor 3 plate reader (Perkin Elmer, Waltham, MA, USA).

#### Cytokine Enzyme-Linked Immunosorbent Assays

Spleen supernatants were analyzed for cytokines according to manufacturer’s instructions: IL-6 (R&D Biosystems, Minneapolis, MN, USA), IL-21 and BAFF (MyBiosource, San Diego, CA, USA). Spleen supernatant was diluted in serum diluent provided for each respective kit. Dilutions used were 15:100 (BAFF), 1:4 (IL-6 and IL-10), and 1:3 (IL-21).

### Statistics

Statistical analyses were performed in GraphPad Prism (La Jolla, CA, USA). All comparisons between two groups were done by Student’s T test with Welch’s correction. Statistical significance is defined as p < 0.05.

## Results

### B Cells From B6.Nba2.BΔIFNAR Mice Fail to Respond to IFNαβ

**Figure 1:**
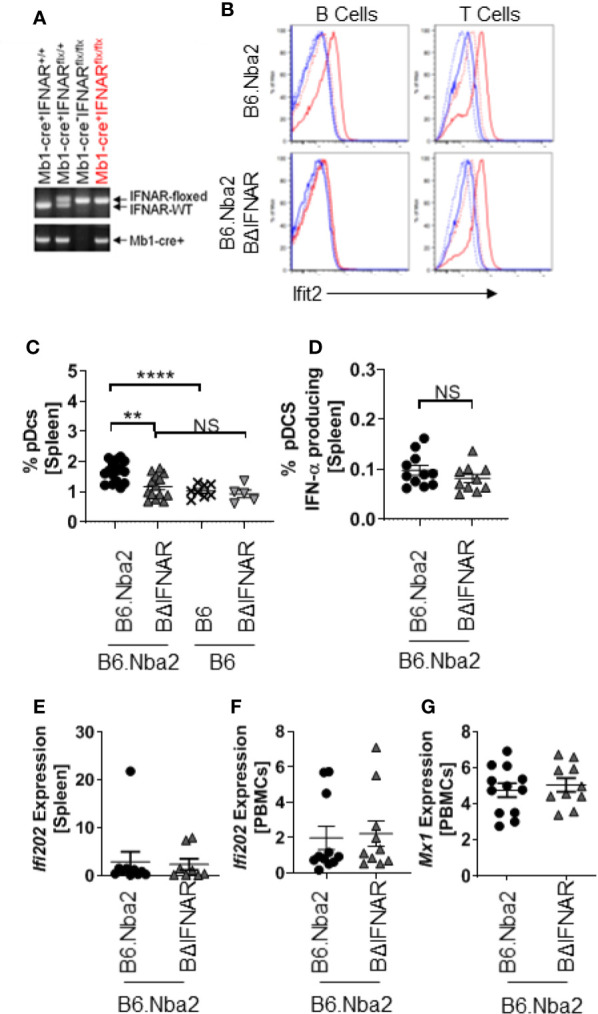
B cells from B6.Nba2.BΔIFNAR mice do not respond to recombinant IFN-α *in vitro*. **(A)** Genotyping by PCR shows the presence of Mb1-cre and floxed IFNAR. **(B)** Primary splenocytes were isolated and incubated overnight, with or without recombinant mouse IFN-αA. Induction of Ifit2 protein was determined in T and B cell populations using flow cytometry. Data shown are representative of four independent analyses. Blue stippled line: No stimulation, control stain; Blue solid line: IFN-α stimulation, control stain; Red stippled line: No stimulation, Ifit2 stained; Red solid line: IFN-α stimulation, Ifit2 stain. **(C, D)** Splenic populations of pDCs **(C)** and SigH+ pDCs **(D)** were quantified in B6.Nba2 (n = 11–17), B6.Nba2.BΔIFNAR (n = 10–14), B6 (n = 8) and B6.BΔIFNAR (n = 5). **(E–G)** Expression of Type-I interferon-induced transcripts was quantified using RT PCR for *Ifi202* **(E, F)** and *Mx1* **(G)** in the spleen and peripheral blood mononuclear cells (PBMCs), respectively. Each symbol represents one mouse and data are shown as Mean ± SEM. **p < 0.01; ****p < 0.0001; Student’s unpaired t-test with Welch’s correction.

To study how IFNαβ stimulation of B cells specifically alters lupus-like disease and contributes to symptom presentation, we created B cell-specific IFNAR-deficient mice (BΔIFNAR) on the lupus-prone B6.Nba2 background (**Figure 1A**). To functionally verify our model we stimulated primary splenocytes with murine recombinant IFN-αA overnight, and measured cell surface markers and intracellular Ifit2, an IFNαβ-induced protein, using flow cytometry (28). B cells from B6.Nba2.BΔIFNAR mice did not show production of Ifit2 in response to IFN-αA stimulation, whereas B cells from WT B6.Nba2 lupus-prone control mice upregulated Ifit2 levels by ~25 fold (**Figure 1B**). This effect was specific to B cells, as both T cells and dendritic cells from B6.Nba2.BΔIFNAR mice upregulated Ifit2 expression after stimulation (**Figure 1B** and data not shown).

### B6.Nba2.BΔIFNAR Mice Express Normal Frequencies of Developing B Cells

To confirm that B6.Nba2.BΔIFNAR mice were not displaying B cell developmental defects, frequencies of early B progenitor cells, pro-B cells, pre-B cells and immature B cells were determined. No differences were identified between B6.Nba2 and B6.Nba2.BΔIFNAR mice (**Supplemental Figure 2**). Interestingly, we observed significantly reduced levels of total splenic pDCs in B6.Nba2.BΔIFNAR mice, however levels of interferon-producing SiglecH+ pDCs were unchanged (**Figures 1C, D**), suggesting that IFNαβ levels would be unaffected. Studies of the expression of interferon stimulated genes (ISGs) in spleen and peripheral blood mononuclear cells (PBMCs) from B6.Nba2 and B6.Nba2.BΔIFNAR mice confirmed that IFNαβ levels were indeed unchanged in these mice (**Figures 1E–G**).

### Serum Anti-Nuclear Autoantibodies Are Reduced in B6.Nba2.BΔIFNAR Mice

**Figure 2:**
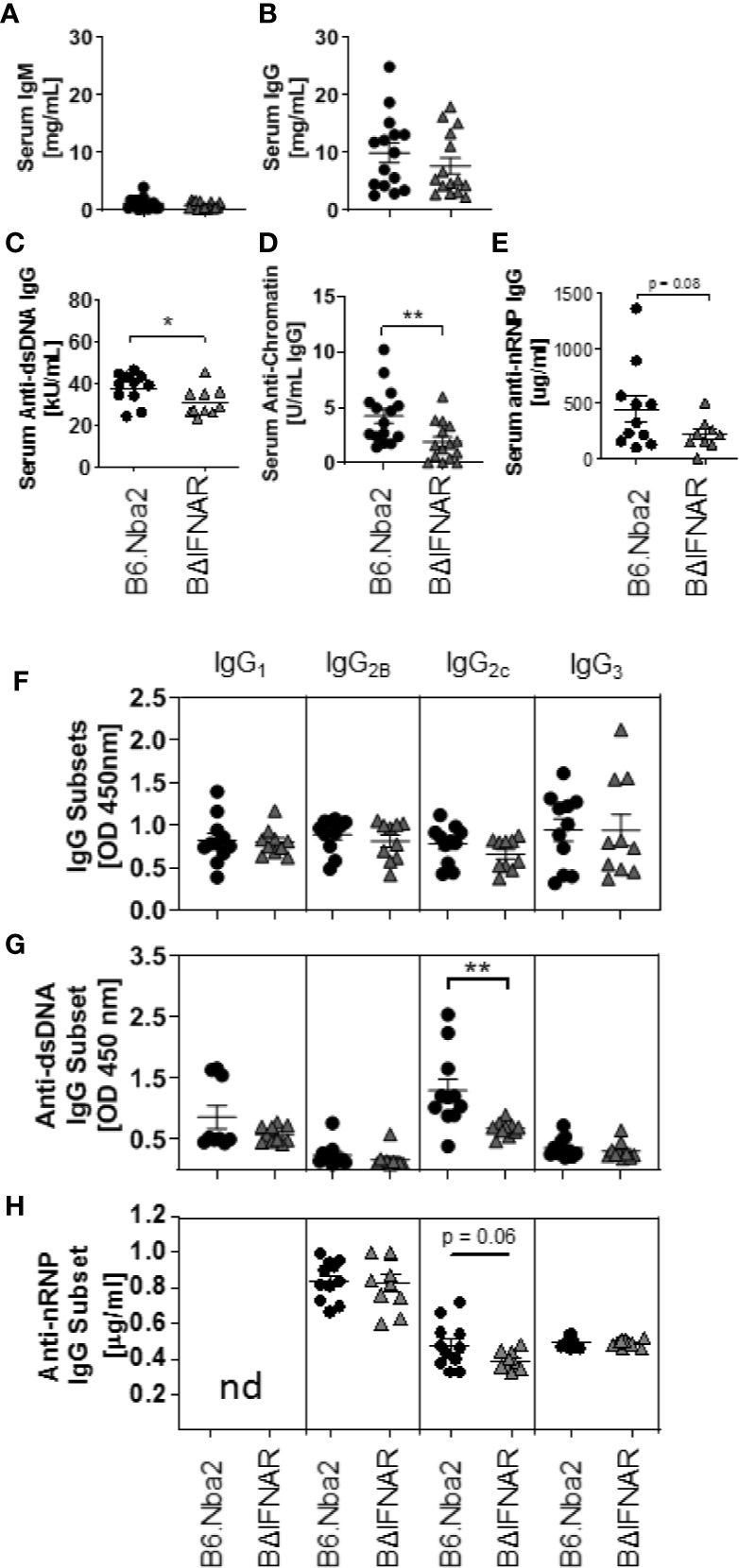
B6.Nba2.BΔIFNAR mice develop reduced ANA levels. **(A–H)** Serum levels of antibodies were measured by ELISA in B6.Nba2 (n = 11–15) and B6.Nba2.BΔIFNAR (n = 10–15): IgM **(A)**, IgG **(B)**, anti-dsDNA IgG **(C)**, anti-chromatin IgG **(D)**, anti-nRNP IgG (**E**), total serum IgG subtypes (IgG1, IgG2b, IgG2c, IgG3) **(F)**, anti-dsDNA IgG subsets **(G)** and anti-nRNP IgG subsets **(H)**. Each symbol represents one mouse and data are shown as Mean ± SEM. *p < 0.05; **p < 0.01. Student T test with Welch’s correction.

Hypergammaglobulinemia and elevated levels of serum ANA are hallmarks of B6.Nba2 lupus-like disease (22, 25). We compared serum antibodies in 4 months old B6.Nba2 and B6.Nba2.BΔIFNAR mice and found no significant differences in the levels of serum IgM (**Figure 2A**), IgG (**Figure 2B**), and IgA (data not shown). In contrast, B6.Nba2.BΔIFNAR mice displayed significantly reduced levels of serum anti-dsDNA IgG, serum anti-chromatin IgG (**Figures 2C, D**) and reduced levels of serum anti-nRNP IgG (**Figure 2E**). There were no differences in total serum IgG subclasses (**Figure 2F**), however, anti-dsDNA and anti-nRNP IgG_2c_ antibodies, but not other IgG subclasses, were decreased in serum of B6.Nba2.BΔIFNAR mice (**Figures 2G, H**).

### Glomerular Immune Complex Deposition and Complement Fixation Are Not Altered in B6.Nba2.BΔIFNAR Mice

Aged B6.Nba2 mice present with a moderate kidney phenotype including IgG-immune complex (IgG-IC) deposition and complement C′3 fixation in the glomeruli, immune cell infiltration, and mesangial cell proliferation (29). We found no difference in mesangial hypercellularity and glomerular area between B6.Nba2.BΔIFNAR mice and B6.Nba2 controls at 4 months of age (**Supplemental Figure 3**). Similarly, we found no difference in the glomerular deposition of IgG- or IgM-IC and no difference in complement C′3 fixation (**Supplemental Figure 3** and data not shown). Surprisingly, we did not also see differences in total IgG2c-IC between B6.Nba2 and B6.Nba2.BΔIFNAR mice (**Supplemental Figure 3**).

### B6.Nba2.BΔIFNAR Mice Display Reduced Splenomegaly and Reduced Activation of B Cells

**Figure 3:**
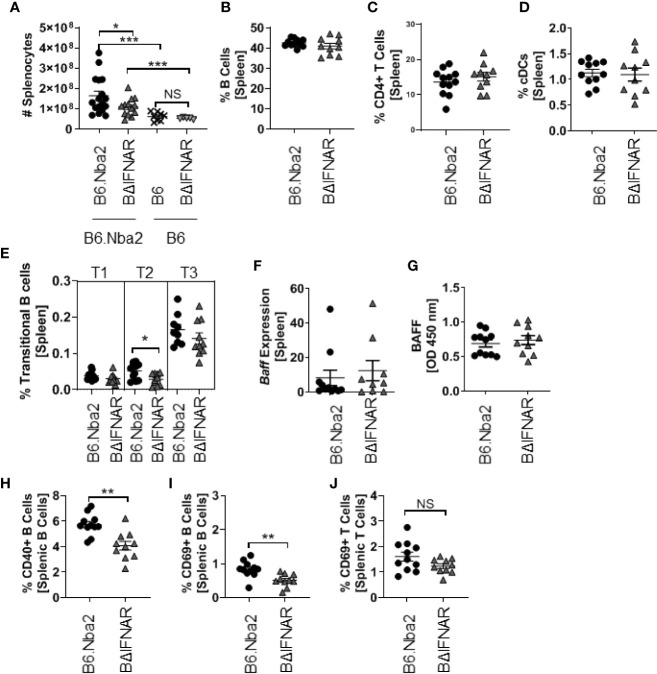
B6.Nba2.BΔIFNAR mice do not develop splenomegaly and display reduced populations of activated B cells. **(A)** Splenomegaly was measured by splenocyte count. **(B–E)** Percentages of total splenic B cells **(B)**, T cells **(C)**, cDCs **(D)**, and Transitional B cell subsets **(E)** were quantified using flow cytometry. **(F, G)** Expression of *Baff* was quantified using RT-PCR and splenic secreted Baff levels were measured by ELISA. **(H–J)** Populations of CD40+ B cells **(H)** and CD69+ activated B cells **(I)** and T cells **(J)** in the spleen were quantified using flow cytometry. Each symbol represents one mouse and data are shown as Mean ± SEM. B6.Nba2: n = 11–17, B6.Nba2.BΔIFNAR: n = 10–13, B6: n=8, B6.BΔIFNAR: n=5. *p < 0.05; **p < 0.01; ***p < 0.001. Student’s unpaired t-test with Welch’s correction.

Splenomegaly, a pronounced presentation of B6.Nba2 lupus-like disease, was quantified and found to be significantly reduced in B6.Nba2.BΔIFNAR mice as compared with B6.Nba2 age-matched mice (**Figure 3A**). Further analyses of the spleen composition, revealed no significant differences in the frequency of total B cells, total T cells, macrophages, conventional DCs (cDC) and neutrophils (**Figures 3B–D** and data not shown). As previously mentioned, total pDCs were significantly reduced in BΔIFNAR mice while the subset of IFNαβ producing pDCs was unchanged (see above, **Figures 1C, D**). Levels of transitional B cells (**Figure 3E**) were similarly unchanged, as were transcriptional levels of *Baff* and splenocyte secreted BAFF (**Figures 3F, G**). Populations of follicular mature B cells and marginal zone B cells also remained unchanged (**Supplemental Figures 4A, B**). However, frequencies of CD69+ and CD40+ B cells were significantly reduced in B6.Nba2.BΔIFNAR mice (**Figures 3H, I**). This was not due to a general reduction in the level of cellular activation, as the CD69+ activated T cell population was unaffected (**Figure 3J**). Thus, expression of IFNAR by B cells in B6.Nba2 mice contributes to splenomegaly and activation of B cells specifically, but alters neither the total B cell nor T cell, macrophage and dendritic cell populations.

### B6.Nba2.BΔIFNAR Mice Display Reduced Levels of Splenic GC B Cells, Memory B Cells, and Plasma Blasts/Plasma Cells

**Figure 4:**
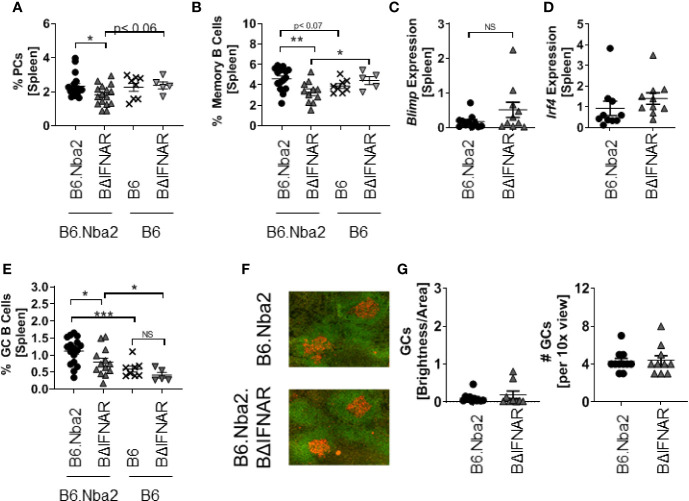
Antibody-secreting cells are reduced in spleens from B6.Nba2 BΔIFNAR mice. **(A, B)** Splenic populations of antibody-producing PB/PCs (CD138+ B220low) **(A)** and memory B cells (B220+CD38+GL7-IgM-) **(B)** were measured by flow cytometry. **(C, D)** Splenic transcripts of *Blimp1* **(C)** and *Irf4* **(D)** were measured by RT PCR. **(E)** The splenic GC B cells (B220+ GL-7+IgM-) were quantified using flow cytometry. **(F)** Immunohistochemistry staining for GCs using B220 (Green) and GL-7 (Red) was imaged. Representative images are shown. **(G)** Mean GC brightness/area was measured and numbers of GCs in a 10x view were enumerated from stains shown in **(F)** B6.Nba2: n = 11–17, B6.Nba2.BΔIFNAR: n = 10–14, B6: n = 8 (B6), B6. BΔIFNAR: n = 5. Each symbol represents one mouse and data are shown as Mean ± SEM. *p < 0.05; **p < 0.01; ***p < 0.001. Student’s unpaired t-test with Welch’s correction. NS: not statistically significant.

Corresponding with the reduced levels of ANAs, B6.Nba2.BΔIFNAR mice displayed significantly reduced levels of plasma blasts/plasma cells (PB/PC) and memory B cell (**Figures 4A, B**). This reduction was specific to the B6.Nba2 model as levels of PB/PC and memory B cells were unchanged between B6 and B6.BΔIFNAR mice (**Figures 4A, B**). It should be noted that the decrease in splenic PB/PC in B6.Nba2.BΔIFNAR mice was not due to an increase in homing to the bone marrow, as bone marrow PB/PC levels were unchanged (**Supplemental Figure 1E**). Interestingly, we found no significant differences in splenic transcript levels of *Blimp* (**Figure 4C**) and *Irf4* (**Figure 4D**) both of which are required for plasma cell differentiation (30–32), suggesting that the differentiation process to PB/PC and memory B cells was unaffected by IFNAR-deficiency.

### B6.Nba2.BΔIFNAR Mice Display Reduced Germinal Center (GC) B Cells With No Change in T Follicular Helper (TFH) Cells

**Figure 5:**
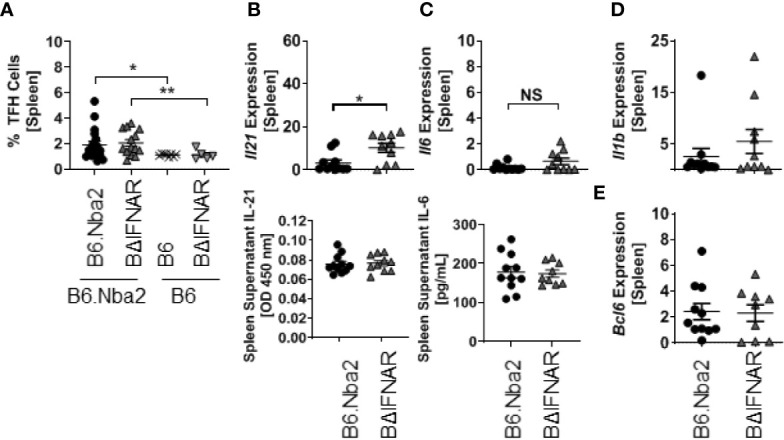
T follicular helper cell populations are not altered in B6.Nba2.BΔIFNAR mice. **(A)** Populations of TFH cells (CD3+ CD4+ PD1+ CXCR5+) were quantified using flow cytometry. **(B, C)** Transcripts and secreted splenic levels of IL-21 and IL-6 were measured. Splenic levels of *Il1b* **(D)** and *Bcl6* **(E)** transcripts were measured using RT PCR. Each symbol represents one mouse and data are shown as Mean ± SEM. B6.Nba2: n = 11, B6.Nba2.BΔIFNAR: n = 10. *p < 0.05; **p < 0.01; Student’s unpaired t-test with Welch’s correction. NS: not statistically significant.

We quantified the GC B cells to determine if the significant decrease in PB/PC and memory B cells was due to reduced GC B cell population. Flow cytometric analyses identified a significant decrease in the GC B cell population of B6.Nba2.BΔIFNAR mice (**Figure 4E**), although immunohistochemistry analyses did not support differences in GC area (**Figures 4F, G**). The reduction in GC B cell numbers, was not due to changes in the TFH cell population (**Figure 5A**) or in levels of IL-6, IL-1β, and IL-21, known to be involved in the GC reaction (**Figures 5B–D**). Similarly, expression levels of *Bcl6*, a crucial transcription factor for TFH and GC B cells (33–35), was unchanged (**Figure 5E**).

### IFNAR-Deficiency Results in Increased Apoptosis of GC B Cells

**Figure 6:**
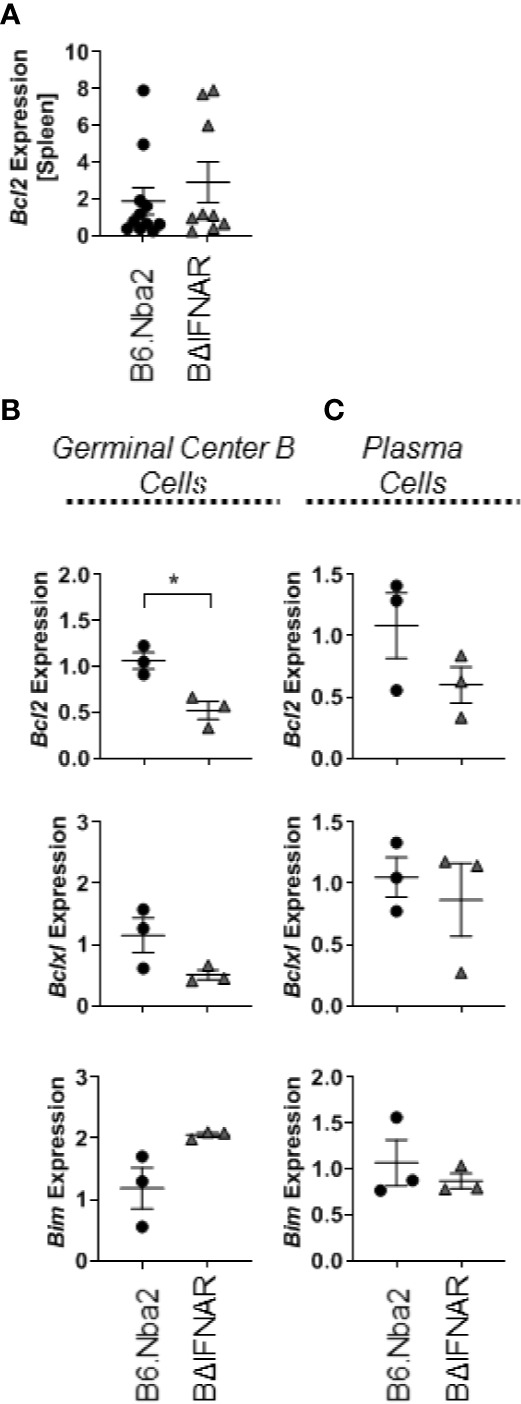
Bcl2 expression is decreased in GC B cells from B6.Nba2.BΔIFNAR mice. Levels of *Bcl2* transcripts were measured in total spleen **(A)**. Transcript levels of *Bcl2*, *Bclxl* and *Bim* were measured on sorted GC B cells **(B)** and sorted PB/PCs **(C)**. Each symbol represents one mouse and data are shown as Mean ± SEM. B6.Nba2: n = 3, B6.Nba2.BΔIFNAR: n = 3. *p < 0.05; Student’s unpaired t-test with Welch’s correction.

IFNα signaling has been found to drive Bcl2 expression in lymphocytes, suggesting that a lack of IFNα signaling may affect B cell survival (36). We evaluated expression of the anti-apoptotic factors *Bcl2* and *Bclxl* in total splenocytes and in sorted GC B cells and PB/PC. While there was no change in expression in total splenocytes (**Figure 6A**), GC B cells from B6.Nba2.BΔIFNAR mice showed decreased expression of *Bcl2* and *Bclxl* (p < 0.05 and p = 0.1428, respectively), and a trend towards elevated expression of *Bim* (p = 0.1202) (**Figure 6B**). In contrast, there were no differences in the expression level of either factor in PB/PC cells (**Figure 6C**). In support of a role for IFNα in regulating Bcl2, splenic B cells from the B6.Nba2 mice showed increased intracellular Bcl2 protein *ex vivo* in response to recombinant IFN-αA stimulation, while B cells from B6.Nba2.BΔIFNAR mice were unable to upregulate intracellular Bcl2 in response to the same conditions (data not shown).

## Discussion

IFNαβ is best known as an anti-viral response (37, 38), but it also plays a significant role in murine and human SLE (4–7, 17–20). This has been shown through increased ISG expression in patients (7–10) and protection from murine lupus-like symptom development in global IFNAR knockouts (17–20, 25). Still, the specific response to IFNαβ by individual immune cells and their relative contribution to disease characteristics during lupus development remains largely unknown. The B6.Nba2 mouse model of lupus express an NZB-derived lupus susceptibility locus (*Nba2*) on distal chromosome 1 and develops spontaneous B and T cell activation and GC formation, elevated PB/PC and autoantibody levels, and IgG-IC deposition in glomeruli (25). We therefore chose this model to study the specific effect of type I interferons on B cell function and pathogenicity during lupus-like disease development. We show here that IFNAR expression by B cells contributes to increased levels of anti-chromatin IgG, anti-dsDNA and anti-nRNP IgG and IgG2c autoantibodies in lupus-prone B6.Nba2 mice, without significantly increasing total levels of IgG or IgM and without skewing total IgG subtype presentation. Similar to the lack of changes in glomerular IgG2c deposition observed in B6.Nba2 global IFNAR knockout mice (17), the reduced levels of anti-dsDNA and anti-nRNP specific IgG2c autoantibodies in the serum of B6.Nba2.BΔIFNAR mice was not accompanied by a decrease in IgG2c deposition in the kidney glomeruli. A few studies have previously described a specific effect of IFNAR signaling in driving immunoglobulin class switching to IgG2c (or IgG2a) (39, 40), however the implication of specific IgG subclass presentation remains unclear in lupus-like disease settings.

Corresponding with reduced levels of anti-chromatin and anti-dsDNA IgG and IgG2c specific autoantibodies, B6.Nba2.BΔIFNAR mice also displayed significantly decreased populations of germinal center B cells, memory B cells and PB/PCs as compared to B6.Nba2 mice, highlighting a role for IFNαβ stimulation to positively affect the germinal center reaction. Surprisingly, we did not observe any difference in GC areas within the spleen. The reason for this is currently unknown, but the fact that other GC cell types, including TFH cells, were unchanged suggest that the process of GC formation was unaltered and the observed effect specific to GC B cells. It should also be noted that levels of GC B cells in B6.Nba2.BΔIFNAR mice remained significantly higher than levels in B6.BΔIFNAR mice, while memory B cells and PB/PCs were actually lower in B6.Nba2.BΔIFNAR mice as compared with B6.BΔIFNAR mice. This suggests that both IFNαβ-dependent and IFNαβ-independent factors drive elevated numbers of GC B cells in B6.Nba2 mice, while IFNαβ is specifically affecting the accumulation of post-GC cells. 

To determine if removal of IFNAR on B cells affected other known mechanisms involved in driving a GC reaction, we analyzed intra-splenic levels of several factors, including IL-6, IL-21, and IL-1β known to be involved in the GC reaction. Of these cytokines, IL-6 and IL-21 were of particular interest, as IL-6 has been associated with increased cell growth and survival in a number of *in vitro* studies (41–44) and affect GC B cells indirectly via differentiation of TFH cells *in vivo (* 45) while IL-21 is known to drive both TFH and GC B cell populations in a virus-infection model (45). Neither IL-6 nor IL-21 were differentially present in spleens of B6.Nba2 and B6.Nba2.BΔIFNAR mice. Furthermore, we did not observe differences in the levels of TFH between the mice, suggesting that the observed changes were due to intrinsic effects in the GC B cells themselves subsequently affecting post-GC B cell populations including memory B cells and PB/PCs.

While IFNαβ alone can directly increase *Bcl2* expression (36, 46), the increased expression of *Bcl2* in GC B cells in the B6.Nba2 model may be compounded by the increased expression of CD40 in the model. CD40 expression by B cells is required for GC formation, Ig isotype class-switching, and sustained production of antibodies, even in the presence of other CD40 expressing APCs (47). Interestingly, *ex vivo* cross-linking of CD40 on human B cells by anti-CD40 specific antibodies also upregulated expression of the anti-apoptotic factor BCL2 in the presence of IL-21 (48), thus directly affecting B cell survival. Similarly, concomitant stimulation of CD40 and the B cell receptor induced increased expression of anti-apoptotic BCL2 family members in human tonsil GC B cells (49), further supporting a potential survival contribution from CD40 expression in B cells. We observed a significant decrease in CD40+ B cells and reduced expression of *Bcl2 and Bclxl* mRNA in GC B cells from B6.Nba2.BΔIFNAR. Based on our data presented here, it is reasonable to hypothesize that in the B6.Nba2 lupus-like model IFNαβ may be increasing B cell activation, leading to increases in CD40+ B cells. CD40 expression and IFNαβ itself may in combination lead to increased survival of GC B cells by increasing pro-apoptotic factor Bcl2. This survival advantage could subsequently contribute to increased differentiation into memory B cells and PB/PCs and elevated production of auto-antibodies. Further studies are needed to establish if altered GC B cell survival is the sole effect of IFNαβ-induced signaling in B cells in this model of lupus.

In summary, in a disease where symptom presentation is extremely heterogeneous, providing effective treatment for each patient means understanding the disease and drivers of symptoms. While we know that blocking IFNAR globally drastically reduces murine lupus-like disease, this approach will also affect IFNαβ responses elicited by viral and/or bacterial infections. In support thereof, Anifrolumab (anti-IFNAR antibody) treatment, while successfully reducing levels of plasma cells (13, 14) and anti-dsDNA antibody levels in a Phase IIb trial (50), resulted in reactivation of herpes simplex virus in a significant number of patients (50). Finally, our studies suggests that therapies that target IFNAR, such as Anifrolumab, or IFNαβ may be more successful in patients who have higher levels of autoantibodies, such as anti-dsDNA antibody levels, or increased B cell activation. Understanding how IFNαβ affect additional cell subsets as these contribute to disease and symptom presentation, may allow us to identify better therapeutic targets, combinatorial treatments or cell-specific therapies to more specifically treat individual patients.

## Data Availability Statement

The original contributions presented in the study are included in the article/**Supplementary Material**, further inquiries can be directed to the corresponding author.

## Ethics Statement

All mice were maintained in the Biological Research Unit at the Lerner Research Institute, in accordance with Cleveland Clinic Foundation Animal Research Committee guidelines. Animal studies were approved by the Institutional Animal Care and Use Committee of the Lerner Research Institute of the Cleveland Clinic Foundation and conducted in compliance with guidelines issued by the National Institutes of Health.

## Author Contributions

EK harvested the mice, and performed all *in vitro* studies, all ELISAs, and all flow cytometry. NP contributed to staining, imaging, and quantification of the kidneys and spleen, as well as genotyping and mouse care. JN blindly scored pathology for H&E and PAS kidney samples. MP contributed to the breeding and genotyping of the mice and to sectioning frozen tissues for immunohistochemistry. TJ designed and guided the study. The manuscript was written by EK and TJ. All authors contributed to the article and approved the submitted version.

## Funding

Funding for this study was provided by NIH R01AI118774 (TNJ).

## Conflict of Interest

The authors declare that the research was conducted in the absence of any commercial or financial relationships that could be construed as a potential conflict of interest.

## Supplementary Material

Supplementary Figure 1 Gating strategy for splenocyte and bone marrow populations shown in figures. **(A)** pDCs in were gated as CD11c+ B220+ and IFN-α producing pDCs were gated as SigH+ PDCA1+. cDCs were gated as B220- CD11c+ **(B)** CD69+ activated cells were determined from B220+ B cells and CD4+ T cells. **(C)** CD40+ activated B cells were gated as IgM+ CD40+ from B220+ B cells. **(D)** Plasma blasts/cells were gated as B220 low CD138+ and then separated by IgD and IgM. **(E)** T follicular helper cells were gated as PD1+ CXCR5+ from the CD3+ CD4+ T cell population. **(F)** Germinal center cells were gated as B220+ GL7+ and IgM- CD38-, while memory cells were gated as B220+ GL7-, then CD38+ IgM-. **(G)** B220+ AA4.1 + cells were separated into transitional B cell populations using IgM and CD23. **(H)** B220+ IgM+ cells were separate into CD23+ CD21- follicular B cells and CD23- CD21+ marginal zone B cells. **(I)** Bone marrow was separated into developmental subsets using B220, CD19, AA4.1, and IgM. **(J)** Plasma blasts/cells were defined as B220 low CD138+, then separated by IgM and IgD expression. All subsets shown were first gated for live cells using FSC SSC pattern.

Supplementary Figure 2 B cell development and bone marrow populations are unchanged on B6.Nba2.BΔIFNAR mice. Flow cytometry was done using the bone marrow from one femur and one tibia per mouse for cell samples. **(A)** B cell development in the bone marrow was analyzed using flow cytometry and percentages of pro B cells, pre-pro B cells pre B cells, and immature B cells were quantified. **(B)** Plasma cells were gated as B220 low CD138+. Each symbol represents one mouse and data are shown as Mean ± SEM. n = 11 (B6.Nba2); n = 10 (B6.Nba2.BΔIFNAR) * p < 0.05; ** p < 0.01; Student’s unpaired t-test with Welch’s correction. NS, not statistically significant.

Supplementary Figure 3 B6.Nba2.BΔIFNAR mice are not protected from glomerulonephritis or immune complex deposition. **(A)** Kidneys from B6.Nba2 and B6.Nba2.BΔIFNAR mice were stained with hematoxylin/eosin (H&E). **(B)** Kidneys from B6.Nba2 and B6.Nba2.BΔIFNAR mice were stained with periodic acid Schiff (PAS). **(C)** Kidneys from B6.Nba2 and B6.Nba2.BΔIFNAR mice were stained with anti-IgG (Red)/anti-C’3 (Green). **(D)** Kidneys from B6.Nba2 and B6.Nba2.BΔIFNAR mice were stained with anti-IgG2c (Red)/anti-C’3 (Green). Stains were used for detection of renal morphology, glomerulonephritis, immune complex deposition and complement fixation. **(E)** Kidneys were scored using both H&E and PAS stains by a blinded pathologist for mesangial hypercellularity. **(F)** Glomerular area was measured and calculated using the H&E staining. **(G, H)** IgG and IgG2c deposition were measured using Image Pro Software and Keyence software respectively. Two sections, representing > 10 glomeruli were analyzed per mouse for each evaluation. Each symbol represents the average score per mouse and data are shown as Mean ± SEM. n = 11 (B6.Nba2); n = 10 (B6.Nba2.BΔIFNAR). * p < 0.05, Student’s unpaired t-test with Welch’s correction.

Supplementary Figure 4 Populations of splenic Marginal Zone B cells and Follicular B cells are unchanged in B6.BΔIFNAR mice. Populations of marginal zone (B220+ CD23 intermediate CD21+ IgM+ and follicular B (B220+ CD21 low CD23+ IgM low) cells were quantified using flow cytometry.
