# Supplementary figures and images for: Partial Protection From Lupus-Like Disease by B-Cell Specific Type I Interferon Receptor Deficiency
Source: Front Immunol. 2021 Jan 8;11:616064. doi: 10.3389/fimmu.2020.616064 (PMC7821742; doi:10.3389/fimmu.2020.616064)

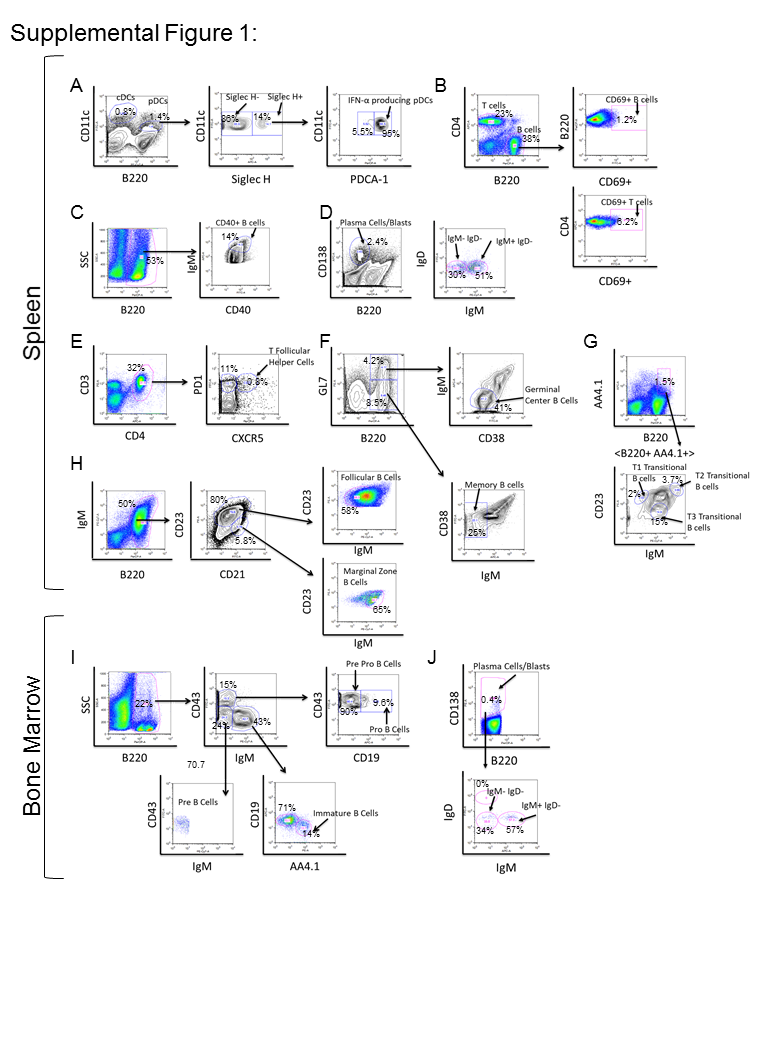

Supplement: Supplementary Figure 1 — Gating strategy for splenocyte and bone marrow populations shown in figures. (A) pDCs in were gated as CD11c+ B220+ and IFN-α producing pDCs were gated as SigH+ PDCA1+. cDCs were gated as B220- CD11c+ (B) CD69+ activated cells were determined from B220+ B cells and CD4+ T cells. (C) CD40+ activated B cells were gated as IgM+ CD40+ from B220+ B cells. (D) Plasma blasts/cells were gated as B220 low CD138+ and then separated by IgD and IgM. (E) T follicular helper cells were gated as PD1+ CXCR5+ from the CD3+ CD4+ T cell population. (F) Germinal center cells were gated as B220+ GL7+ and IgM- CD38-, while memory cells were gated as B220+ GL7-, then CD38+ IgM-. (G) B220+ AA4.1 + cells were separated into transitional B cell populations using IgM and CD23. (H) B220+ IgM+ cells were separate into CD23+ CD21- follicular B cells and CD23- CD21+ marginal zone B cells. (I) Bone marrow was separated into developmental subsets using B220, CD19, AA4.1, and IgM. (J) Plasma blasts/cells were defined as B220 low CD138+, then separated by IgM and IgD expression. All subsets shown were first gated for live cells using FSC SSC pattern. [file Image_1.tif]

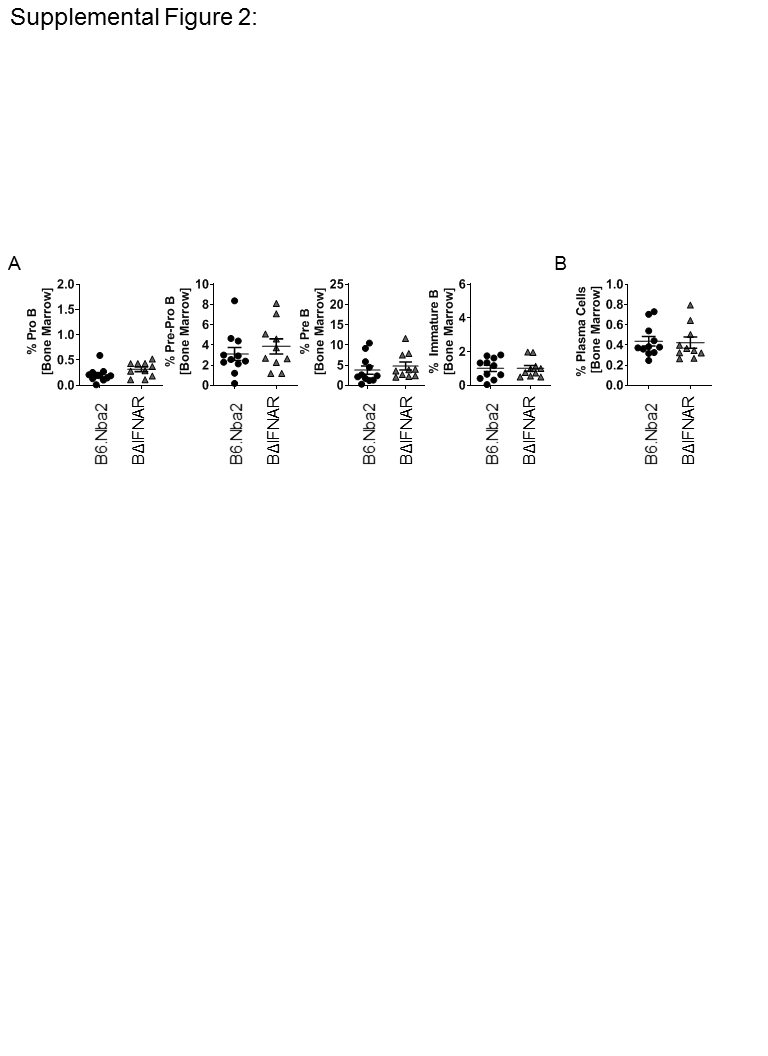

Supplement: Supplementary Figure 2 — B cell development and bone marrow populations are unchanged on B6.Nba2.BΔIFNAR mice. Flow cytometry was done using the bone marrow from one femur and one tibia per mouse for cell samples. (A) B cell development in the bone marrow was analyzed using flow cytometry and percentages of pro B cells, pre-pro B cells pre B cells, and immature B cells were quantified. (B) Plasma cells were gated as B220 low CD138+. Each symbol represents one mouse and data are shown as Mean ± SEM. n = 11 (B6.Nba2); n = 10 (B6.Nba2.BΔIFNAR) * p < 0.05; ** p < 0.01; Student’s unpaired t-test with Welch’s correction. NS, not statistically significant. [file Image_2.tif]

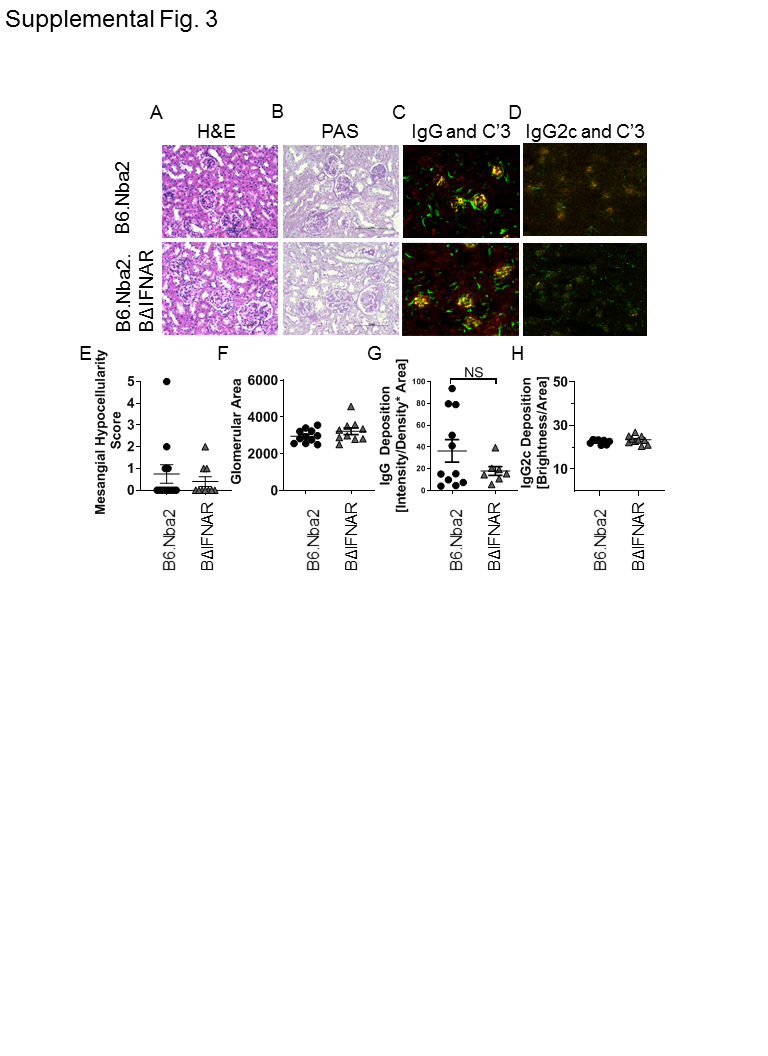

Supplement: Supplementary Figure 3 — B6.Nba2.BΔIFNAR mice are not protected from glomerulonephritis or immune complex deposition. (A) Kidneys from B6.Nba2 and B6.Nba2.BΔIFNAR mice were stained with hematoxylin/eosin (H&E). (B) Kidneys from B6.Nba2 and B6.Nba2.BΔIFNAR mice were stained with periodic acid Schiff (PAS). (C) Kidneys from B6.Nba2 and B6.Nba2.BΔIFNAR mice were stained with anti-IgG (Red)/anti-C’3 (Green). (D) Kidneys from B6.Nba2 and B6.Nba2.BΔIFNAR mice were stained with anti-IgG2c (Red)/anti-C’3 (Green). Stains were used for detection of renal morphology, glomerulonephritis, immune complex deposition and complement fixation. (E) Kidneys were scored using both H&E and PAS stains by a blinded pathologist for mesangial hypercellularity. (F) Glomerular area was measured and calculated using the H&E staining. (G, H) IgG and IgG2c deposition were measured using Image Pro Software and Keyence software respectively. Two sections, representing > 10 glomeruli were analyzed per mouse for each evaluation. Each symbol represents the average score per mouse and data are shown as Mean ± SEM. n = 11 (B6.Nba2); n = 10 (B6.Nba2.BΔIFNAR). * p < 0.05, Student’s unpaired t-test with Welch’s correction. [file Image_3.tif]

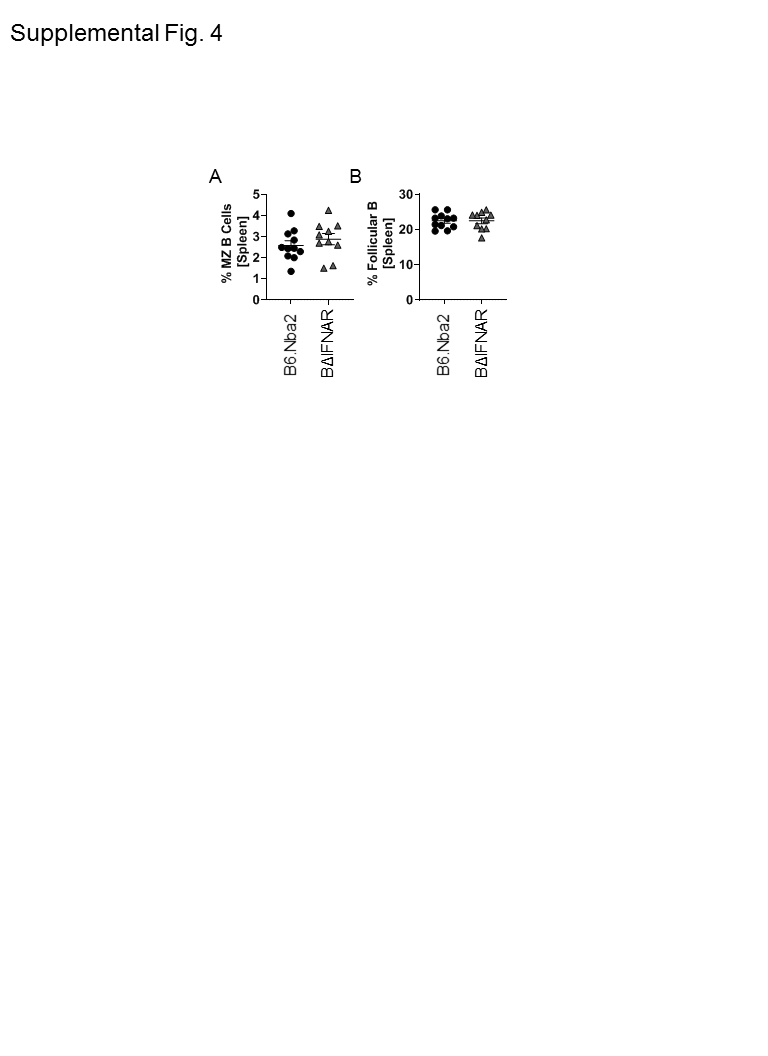

Supplement: Supplementary Figure 4 — Populations of splenic Marginal Zone B cells and Follicular B cells are unchanged in B6.BΔIFNAR mice. Populations of marginal zone (B220+ CD23 intermediate CD21+ IgM+ and follicular B (B220+ CD21 low CD23+ IgM low) cells were quantified using flow cytometry. [file Image_4.tif]
